# Optimal Obesity- and Lipid-Related Indices for Predicting Metabolic Syndrome in Chronic Kidney Disease Patients with and without Type 2 Diabetes Mellitus in China

**DOI:** 10.3390/nu14071334

**Published:** 2022-03-23

**Authors:** Hangtian Li, Qian Wang, Jianghua Ke, Wenwen Lin, Yayong Luo, Jin Yao, Weiguang Zhang, Li Zhang, Shuwei Duan, Zheyi Dong, Xiangmei Chen

**Affiliations:** 1 National Clinical Research Center for Kidney Diseases, State Key Laboratory of Kidney Diseases, Beijing Key Laboratory of Kidney Disease Research, First Medical Center of Chinese PLA General Hospital, Nephrology Institute of the Chinese People’s Liberation Army, Beijing 100853, China; hangtianli2022@163.com (H.L.); wangqian@301hospital.com.cn (Q.W.); jianghuak@126.com (J.K.); linwenwen9999@163.com (W.L.); lyayong321@163.com (Y.L.); jin_yao1120@163.com (J.Y.); weiguangzhang1@163.com (W.Z.); zhang301li@163.com (L.Z.); shuweiduan@163.com (S.D.); 2 School of Clinical Medicine, Guangdong Pharmaceutical University, Guangzhou 510006, China

**Keywords:** metabolic syndrome, type 2 diabetes mellitus, chronic kidney disease, visceral fat area, visceral adiposity index, lipid accumulation product

## Abstract

Existing obesity- and lipid-related indices are inconsistent with metabolic syndrome (MetS) in chronic kidney disease (CKD) patients. We compared seven indicators, including waist circumference (WC), body mass index (BMI), visceral fat area (VFA), subcutaneous fat area (SFA), visceral adiposity index (VAI), Chinese VAI and lipid accumulation product (LAP), to evaluate their ability to predict MetS in CKD patients with and without Type 2 diabetes mellitus (T2DM) under various criteria. Multivariate logistic regression analysis was used to investigate the independent associations between the indices and metabolic syndrome among 547 non-dialysis CKD patients, aged ≥18 years. The predictive power of these indices was assessed using receiver operating characteristic (ROC) curve analysis. After adjusting for potential confounders, the correlation between VAI and MetS was strongest based on the optimal cut-off value of 1.51 (sensitivity 86.84%, specificity 91.18%) and 2.35 (sensitivity 83.54%, specificity 86.08%), with OR values of 40.585 (8.683–189.695) and 5.076 (1.247–20.657) for males and females with CKD and T2DM. In CKD patients without T2DM, based on the optimal cut-off values of 1.806 (sensitivity 98.11%, specificity 72.73%) and 3.11 (sensitivity 84.62%, specificity 83.82%), the OR values were 7.514 (3.757–15.027) and 3.008 (1.789–5.056) for males and females, respectively. The area under ROC curve (AUC) and Youden index of VAI were the highest among the seven indexes, indicating its superiority in predicting MetS in both male and female CKD patients, especially those with T2DM.

## 1. Introduction

Metabolic syndrome (MetS) is a group of syndromes characterized by cardiometabolic risks, including central obesity, elevated blood pressure, dysglycemia, elevated triglyceride levels and low high-density lipoprotein cholesterol (HDL-C) levels [1]. As of 2017, the prevalence of metabolic syndrome was approximately 15.5% in China [2]. MetS is not only associated with a high risk of coronary heart disease, stroke and cardiovascular death [3], but is also strongly associated with chronic kidney disease [4].

Chronic kidney disease (CKD) is a global public health problem due to its high morbidity and mortality [5,6]. The global prevalence of CKD is between 11–13% [7], and China ranks first worldwide [8] with 132 million CKD patients. Related literature reports that patients with MetS not only have a doubled risk of CKD and microalbuminuria [9], but also have accelerated progression of CKD [10]. Type 2 diabetes mellitus (T2DM) is the leading cause of CKD, and a significant comorbidity that leads to non-diabetic nephropathy. CKD patients with T2DM are a special risk group, as they have a higher mortality rate and a higher risk of hypoglycemia and metabolic syndrome than general CKD patients [11]. The increased incidence of obesity-related glomerular disease (ORG) is associated with the global obesity epidemic, and perirenal fat (a type of visceral fat) may be a typical biomarker for it [12]. Therefore, early identification of high-risk MetS patients is necessary to prevent the occurrence and development of MetS and CKD and for the Asian obesity phenotype [13].

A number of studies have found that various obesity- and lipid-related parameters are highly correlated with MetS, which can be used to predict MetS. Body Mass Index (BMI) is traditionally the most widely used measure of obesity, but it fails in differentiating lean and fat masses [14]. Waist Circumference (WC) is an important indicator for evaluating central obesity, but it cannot distinguish visceral fat from subcutaneous fat [15]. The distribution of body fat is a well-known determinant of metabolic risk, and the accumulation of visceral fat is thought to be a major correlate of metabolic syndrome [16]. Lipid accumulation product (LAP), the visceral adiposity index (VAI) and the Chinese visceral adipose index (CVAI) are three recent indicators used to estimate visceral adiposity; CVAI is a reliable and applicable indicator established in the Chinese population to assess visceral fat dysfunction [17]. However, no study has reported that these seven parameters have been studied in patients with CKD, and the parameters that are more appropriate for MetS risk remain controversial.

We hypothesized that a simple, clinically measurable alternative could be used to identify MetS in people with CKD, especially those with type 2 diabetes. Therefore, our aim was to compare the predictive power of these 7 “optimal” obesity- and lipid-related parameters to identify MetS in Chinese male and female CKD patients with and without T2DM, based on different criteria.

## 2. Materials and Methods

### 2.1. Study Design and Participant

This cross-sectional study included 570 patients with CKD who were hospitalized in the Department of Nephrology of the First Medical Center of the Chinese People’s Liberation Army General Hospital between March and November 2021. Participants aged ≥18 years, diagnosed with CKD, and those with no missing biochemical measurements were included. Pregnant women, patients who are on dialysis (including hemodialysis and peritoneal dialysis), patients who are unable to perform visceral fat area testing due to multiple serous effusions and patients who omitted WC measurements were excluded. Finally, 547 non-dialysis CKD patients with anthropometric and clinical examination information were included in the analysis (Supplementary Figure S1). This study was carried out in accordance with the Declaration of Helsinki, and approved by the Ethics Committee of the Chinese People’s Liberation Army General Hospital. The ethical approval number was S2017-133-01. All the participants provided signed informed content and agreed to attend this survey.

The information collected in this study included sociodemographic characteristics, medical history, family history, laboratory tests and visceral fat area. All practicing physicians who have been trained by Good Clinical Practice (GCP) and have passed the consistency evaluation assessment, collect and encode the clinical trial electronic data capture system (EDC) platform, and retrieve it after review and inspection by professionals. Anthropometric measurements, including weight, height, waist circumference, blood pressure and visceral fat area, were measured by professional researchers following standard protocols. The patients were asked to wear light clothing, and were barefoot when the weight and height were measured. Waist circumference was measured using a flexible plastic tape measure around the navel level after the patient exhaled. Visceral fat area and subcutaneous fat area were measured using an Omron Visceral Fat Detection Device (Model: DHS-2000, OMRON HEALTHCARE Co.ltd, Matsusaka, Japan). Blood pressure in the patient’s non-dominant arm was measured using automated electronic equipment, and after a 5-min rest, blood pressure was measured three times with a one-minute interval. The mean systolic and diastolic blood pressures of the three readings were recorded using a questionnaire. BMI was calculated as weight divided by height squared. The calculation of VAI [18], CVAI [17] and LAP [19] are as follows:

1. Male: **VAI** = WC (cm)/[39.68 + 1.88 × BMI (Kg/m^2^)] × [TG (mmol/L)/1.03] × [1.31/HDL (mmol/L)]

**CVAI** = −267.93 + age (y) + 0.03 × BMI (Kg/m^2^) + 4.00 × WC (cm) + 22.00 × Lg TG (mmol/L) − 16.32 × HDL (mmol/L)

**LAP** = [WC (cm) − 65] × TG (mmol/L)

2. Female: **VAI** = WC (cm)/[36.58 + 1.89 × BMI (Kg/m^2^)] × [TG (mmol/L)/0.81] × [1.52/HDL (mmol/L)]

**CVAI** = −187.32 + age (y) + 4.32 × BMI (Kg/m^2^) + 1.12 × WC (cm) + 39.76 × Lg TG (mmol/L) − 11.66 × HDL (mmol/L)

**LAP** = [WC (cm) − 58] × TG (mmol/L)

### 2.2. Biochemical Measurements

Venous blood was collected on the morning of the second day of hospitalization after the patient had fasted for 8–10 h, in order to determine fasting blood glucose (FBG), uric acid (UA), creatinine (Cr), total cholesterol (TC), triglyceride (TG), high-density lipoprotein cholesterol (HDL-C), low-density lipoprotein cholesterol (LDL-C) and other biochemical indicators. The estimated glomerular filtration rate (eGFR) was calculated using the Chronic Kidney Disease Epidemiology Collaboration (CKD-EPI) equation [20].

### 2.3. Definition of Variables

According to international guidelines [21], CKD is determined if one or both of the following criteria were met for a minimum period of 3 months: (1) GFR < 60 mL/min/173 m^2^ (2) markers of kidney damage (1 or more); and urinary sediment abnormality, abnormalities on histology and history of kidney transplantation.

T2DM was defined as previously diagnosed diabetes mellitus, use of insulin or hypoglycemic drugs, or fasting blood glucose ≥ 7.0 mmol/L.

MetS was identified by the Chinese Guidelines for the Prevention and Treatment of Type 2 Diabetes (2020 Edition) [22], the revised National Cholesterol Education Program Adult Treatment Group (ATPIII) [23] and the International Diabetes Federation (IDF) [24]. The diagnostic criteria in China (2020 edition), wherein three or more can be considered MetS, are as follows: (1) abdominal obesity (central obesity): WC ≥ 90 cm for men and ≥85 cm for women; (2) hyperglycemia: fasting blood glucose ≥ 6.1 mmol/L or 2 h blood glucose after sugar load ≥ 7.8 mmol/L and those who have been diagnosed with diabetes and treated; (3) hypertension: blood pressure ≥ 130/85 mmHg (1 mmHg = 0.133kPa) and (or) confirmed hypertension and treated; and (4) fasting triglycerides (TG) ≥ 1.70 mmol/L, (5) fasting HDL-C < 1.04mmol/L. According to the definition of NCEP-ATPIII, MetS requires at least 3 of 5 components: (i) central obesity (WC: ≥90 cm in men and ≥80 cm in women); (ii) elevated TG (TG ≥ 1.7 mmol/L); (iii) low HDL-C (HDL-C: <1.03 mmol/L in men, <1.29 mmol/L in women); (iv) elevated blood pressure (systolic/diastolic ≥ 130/85 mmHg, or use antihypertensive drugs); and (v) hyperglycemia (FPG ≥ 5.6 mmol/L or previously diagnosed with diabetes). IDF is based on central obesity, and has four components: (1) elevated blood pressure; (2) elevated TG; (3) low HDL cholesterol; and (4) high blood sugar.

### 2.4. Statistical Analysis

Data were tested for normality and homogeneity of variance before performing statistical analysis. Normally distributed measurement data were expressed as mean ± standard deviation (SD), while non-normally distributed data were expressed as the median of the interquartile range (IQR) (25%, 75%). Comparisons between groups were performed using Student’s *t*-test, chi-square test or Mann-Whitney U test. Binary logistic regression analysis was used to determine the relationship between obesity- and lipid-related indices and the incidence of MetS. Data were summarized as odds ratios (ORs) and regression coefficients (95% CIs). Adjusted variables were diagnosed by collinearity, and the diagnosis was based on the following criteria: variance inflation factor (VIF) > 10 or tolerance around 0.1; condition index > 30; and variance ratio > 50%. Select variables are not collinear. Considering possible complete separation or quasi-complete separation (complete separation, quasi-complete separation), Firth’s bias-reducing penalized likelihood method was used for analysis.

When performing binary logistic regression, adjustments were made for patients’ age, systolic blood pressure, total cholesterol, low-density lipoprotein (LDL) cholesterol and eGFR. Receiver operating characteristic (ROC) curve analysis was used to compare the diagnostic performance of obesity- and lipid-related indices for MetS. All statistical analyses were performed using IBM SPSS statistical software, version 25 (IBM Corporation, Armonk, New York, NY, USA). Differences were considered statistically significant at *p* values (two-sided) of <0.05.

## 3. Results

### 3.1. General Characteristics of Patients with CKD

A total of 537 CKD patients participated in the basic analysis, including 372 male patients with an average age of 49.64 ± 13.78 years and 165 female patients with an average age of 51.96 ± 12.46 years. The prevalence of T2DM was 57.5% in males and 42.4% in females; while the prevalence of MetS in CKD patients with T2DM was 80.4% in males and 84.3% in females; and the prevalence of MetS in patients with CKD without T2DM was 50% in males and 27.4% in females.

### 3.2. Different Characteristics of Obesity- and Lipid-Related Indices in Male and Female CKD Patients with and without T2DM

Table 1 and Table 2 show the sociodemographic and general characteristics of male and female patients in this study. Participants were divided into four groups according to the presence or absence of T2DM and the presence or absence of MetS. In CKD patients with T2DM, obesity- and lipid-related parameters (BMI, WC, VFA, SFA, VAI, CVAI, LAP) and clinical indicators (DBP, TG, HDL-C) were significantly increased (all *p* < 0.05). In addition, only female patients with MetS had significantly higher SBP than those without MetS (*p* < 0.05), while there were no significant differences in other indicators between men and women (*p* > 0.05). Obesity and lipid-related parameters (BMI, WC, VFA, SFA, VAI, CVAI and LAP), clinical indicators (SBP, TG, HDL-C and creatinine) in MetS men and women in CKD patients without T2DM were higher than those without MetS (all *p* < 0.05), and only male patients with MetS had significantly higher DBP and FBG levels than those without MetS (*p* < 0.05), while there was no significant difference in other indicators between men and women (*p* > 0.05).

### 3.3. Association of Obesity- and Lipid-Related Indices with Prevalence of MetS

According to the optimal cutoff value, BMI, WC, VFA, SFA, VAI, CVAI and LAP were significantly associated with MetS in all criteria. After adjusting for age, systolic blood pressure, total cholesterol, low-density lipoprotein cholesterol and eGFR, using the criteria of the Chinese Guidelines for the Prevention and Treatment of Type 2 Diabetes (2020 Edition), the OR value of VAI was the largest among male and female CKD patients with T2DM, being 40.585 (95% CI 8.683–189.695) and 5.076 (95% CI 1.247–20.657), respectively. The OR value of VAI was also the largest among male and female CKD patients without T2DM, being 7.514 (95% CI 3.757–15.027) and 3.008 (95% CI 1.789–5.056), respectively (Table 3). After adjusting for age, systolic blood pressure, total cholesterol, LDL cholesterol and eGFR, using the NCEP-ATP III criteria, the OR value of VAI was the highest in males and females with CKD and T2DM, being 71.795 (95% CI 14.460–356.467) and 1.749 (95% CI 1.083–2.824), the OR values for VAI were also the highest in men and women with CKD without T2DM, at 4.055 (95% CI 2.392–6.874) and 6.322 (95% CI 2.665–14.996), respectively (Supplementary Table S1). After adjusting for age, systolic blood pressure, total cholesterol, LDL cholesterol and eGFR, using the IDF criteria, WC had the highest, with OR of 2.733 (95% CI 1.797–4.156) in men with CKD and T2DM, but in women with CKD and T2DM, while the OR values of BMI were the largest, at 2.999 (95% CI 1.653–5.441). The OR values of BMI were the largest in men and women with CKD without T2DM, being 2.146 (95% CI 1.672–2.756) and 1.619 (95% CI 1.317–1.990), respectively (Supplementary Table S2).

### 3.4. Receiver Operating Characteristic (ROC) Curve Analysis

**Figure 1:**
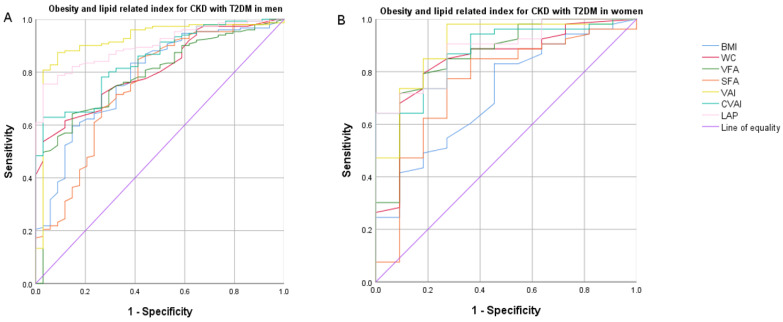

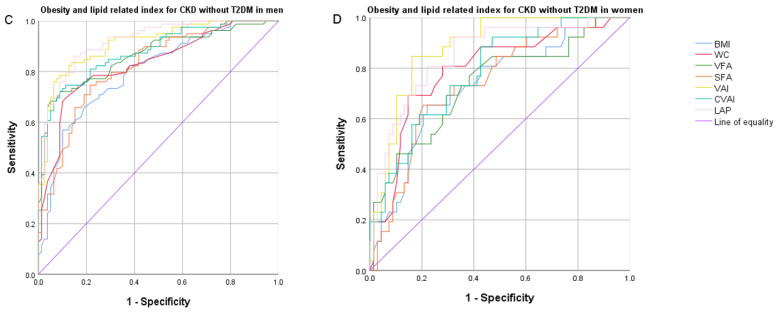
Comparison of the diagnostic value of BMI, WC, VFA, SFA, VAI, CVAI and LAP in predicting metabolic syndrome using Chinese (2020) criteria in CKD men and women with and without T2DM. (**A**) obesity and lipid related index for CKD with T2DM in men; (**B**) obesity and lipid related index for CKD with T2DM in women; (**C**) obesity and lipid related index for CKD with T2DM in men; (**D**) obesity and lipid related index for CKD with T2DM in women; BMI, body mass index; WC, waist circumference; VFA, visceral fat area; SFA, subcutaneous fat area; VAI, visceral adiposity index; CVAI, Chinese visceral adiposity index; LAP, lipid accumulation product; OR, odds ratio; CKD, chronic kidney disease; T2DM, Type 2 diabetes mellitus.

Table 4 shows the diagnostic ability of BMI, WC, VFA, SFA, VAI, CVAI and LAP for MetS in male and female CKD patients with or without T2DM under different criteria through ROC curve analysis. In the China Guidelines for the Prevention and Treatment of Type 2 Diabetes (2020 Edition) diagnostic criteria for metabolic syndrome (Figure 1), we observed that in CKD patients with and without T2DM, the area under the curve of BMI, WC, VFA, SFA, VAI, CVAI and LAP in MetS patients was significantly different between males and females (*p* < 0.05). Among them, the areas under the ROC curve of VAI in men and women CKD patients with T2DM were the largest, at 0.920 (*p* < 0.001) and 0.902 (*p* < 0.001), respectively. The maximum cut-off value of Youden’s index for men was 1.51, with a sensitivity of 86.84% and a specificity of 91.18%; the maximum cut-off value of Youden’s index for women was 1.806, with a sensitivity of 98.11% and a specificity of 72.73%. Among CKD patients without T2DM, the area under the ROC curve of LAP was the largest in male patients, but there was no significant difference with VAI, at 0.921 vs. 0.910 (*p* < 0.001), and the area under the ROC curve of VAI was the largest in female patients.

Supplementary Figures S2 and S3 show the diagnostic ability of obesity- and lipid-related indices for MetS in male and female CKD patients with or without T2DM in the NECP-ATP III and IDF criteria, respectively. In the NECP-ATPIII diagnostic criteria for MetS, the area under the ROC curve of VAI was the largest among the four subgroups, which were 0.957, 0.965, 0.895 and 0.921, respectively (*p* < 0.001). In the diagnostic criteria for IDF MetS, except for male patients without T2DM, the area under the ROC curve of CVAI was the largest at 0.980 (*p* < 0.001) and the area under the ROC curve of the other subgroups of WC was the largest, being 0.998, 1, 0.923 (*p* < 0.001), respectively.

## 4. Discussion

This study investigated the value of commonly used obesity- and lipid-related parameters, including BMI, WC, VFA, SFA, VAI, CVAI and LAP in identifying different criteria for MetS in the CKD population. Our results show that these parameters have reliable predictive accuracy for the diagnosis of MetS, according to the criteria of China (2020) and ATP III. Based on our findings, the VAI may outperform other parameters in predicting MetS. Among the IDF criteria, BMI had the best performance in diagnosing MetS. This is the first report to analyze and compare the use of different criteria to predict obesity- and lipid-related parameters of MetS in Chinese patients with CKD based on the presence and absence of T2DM.

MetS has become a global problem with the influence of Western lifestyles, including fast food consumption and reduced physical activity due to a sedentary work culture [25,26], and its incidence is even higher in some developing countries. According to data, as of 2010, the prevalence of metabolic syndrome among Chinese adults was estimated to be at 33.9%, and was gradually increasing, indicating that approximately 454 million adults in China may be at high risk of cardiovascular disease and diabetes [27]. Since components of MetS are traditional risk factors for CKD and CKD-related cardiovascular disease, corresponding disease interventions may also benefit the kidneys [28]. With such a number of MetS population in China, finding a simple and effective MetS diagnostic marker is crucial for clinical screening and prevention.

The MetS definition of the International Diabetes Federation (IDF) and the ATPIII standard updated by the American Heart Association/American Heart, Lung and Blood Association (AHA/NHLBI) in 2005 are the most recognized and widely used standards in the world, and are often used in China, particularly in clinical trials and research. Our China Guidelines for the Prevention and Treatment of Type 2 Diabetes (2020 Edition) diagnostic criteria for MetS, which cut-off points for WC, HDL-C and hyperglycemia differed from IDF and NECP-ATPIII, may be more suitable for the Chinese population. In addition, an update to this guideline is already being published in 2022. According to this latest guideline [29], the elderly population (≥60 years old) in the country reached 260.4 million in 2020, of which 30% of the elderly suffer from diabetes, and T2DM accounts for more than 9–5%. From the perspective of prevention, early diagnosis of metabolic syndrome is very necessary to prevent the occurrence and development of T2DM, as it is a pathology that increases with age. Among them, for the IDF criteria, central obesity was a necessary condition, while for the other two criteria, the other four conditions were equally important for the diagnosis of MetS as central obesity.

Obesity, especially central obesity and T2DM, is an important part of the diagnosis of MetS. The occurrence of MetS in people with central obesity may be closely related to an increase in visceral adipose tissue, a decrease in subcutaneous tissue expansion and the related metabolic changes caused by ectopic storage of triglycerides in different organs [30]. Obesity and metabolic syndrome are common in patients with T2DM, and CKD patients with T2DM have a higher incidence of MetS and a greater risk of cardiovascular disease than patients with CKD without T2DM [31]. Compared with BMI, a traditional measure of whole-body adiposity, WC is more suitable for evaluating central adiposity, but it cannot be used to distinguish visceral adipose tissue from subcutaneous adipose tissue [32]. Excess visceral fat is associated with a higher cardiometabolic risk than subcutaneous fat or a high BMI. In other words, people with excess visceral fat may be more likely to develop MetS and cardiovascular disease, which is similar to visceral fat. The accumulation of adipocytokines leads to dysregulation of adipocytokine production and secretion, and the reduction of adiponectin is a direct mechanism for the occurrence of MetS and cardiovascular disease [33]. In our study, visceral fat area was better than a subcutaneous fat area for diagnosing MetS in CKD patients with and without T2DM (AUC, 0.785 vs. 0.75, 0.860 vs. 0.754, 0.865 vs. 0.860) and more relevant with MetS occurrence risk (OR, 1.021 vs. 1.019, 1.066 vs. 1.020, 1.051 vs. 1.027, 1.037 vs. 1.011). LAP is an index calculated based on WC and serum triglyceride levels. In previous studies, which included 247 kidney disease outpatients [34], LAP was found to be the best predictor of MetS in patients with CKD stages 3–5 and maintenance hemodialysis (MHD), and found that LAP had the highest AUC level in predicting MetS among the six indicators (LAP, VAI, BMI, WC, WHtR and WHR) (male, 0.908; female, 0.864). In a study of 1603 MHD patients (54.6 ± 16 years old) in Guizhou Province, China [35], when LAP, BMI, WHtR, CI, BRI, ABSI, LAP and VAI were compared, LAP was the most accurate indicator for diagnosing MetS (male, 0.88; female, 0.87). In this study, the diagnostic performance of LAP was second only to that of VAI in both China (2020) and NECP-ATP III criteria. In the subgroup, LAP predicted MetS with the highest AUC of 0.921 (China 2020 criteria, male CKD patients without T2DM), the lowest AUC was 0.848 (NECP-ATP III criteria, male CKD patients without T2DM). VAI and CVAI scores were calculated based on WC, BMI, TG and HDL in 315 Caucasians (BMI, 20–30 kg/m^2^) and 485 Chinese people. It has been reported in the literature [36] that CVAI has the strongest correlation with the prevalence of CVD and CKD in T2DM patients, and compared with NC, WC, WHR, LAP and VAI, CVAI in male patients, was associated with a higher prevalence of CVD and CKD (OR, 1.35, 1.38), as well as in female patients (OR, 1.32, 2.5). We did not find an optimal diagnostic performance of CVAI for MetS in any population. VAI has been shown to have excellent predictive ability in many studies. A study by Dongxue Dai et al. [37] on rural populations in Northeast China found that VAI and LAI were significantly correlated with CKD, and were significantly better than BMI and WC in predicting CKD in female patients. This result was also confirmed in the Taiwanese population [38], wherein a higher VAI score had a higher risk of CKD. This coincides with our study, which showed that VAI has an excellent predictive ability for MetS in patients with CKD. One of the key reasons why VAI is superior to other measures, including body weight/BMI, in CKD patients may be due to severe sarcopenia in CKD patients, which is indistinguishable from body fat in body weight measurements [39]. It is established that patients with MetS have a doubled risk of CKD; therefore, VAI can be a good predictive tool for MetS in patients with CKD.

This study has certain limitations. First, our study had a cross-sectional design and we could not identify causality, and thus, we will follow up with the patients further. Second, the surveyed population was Chinese and aged ≥18 years, and caution should be exercised when generalizing the results to other ethnicities. Third, details about long-term medication use, education and health status were not recorded in this study, which may have influenced the results. Finally, the lack of fasting insulin in this study makes it difficult to analyze information on insulin resistance.

## 5. Conclusions

Our study indicated that VAI is an important risk factor for T2DM in Chinese patients with CKD. In CKD patients, especially those with T2DM – superior to that of BMI, WC, VFA, SFA, CVAI and LAP–VAI demonstrated the best predictive power for MetS based on the Youden index in both sexes.

## Figures and Tables

**Table 1:** Comparison of clinical characteristics of male patients according to the Chinese Guidelines for the Prevention and Treatment of Type 2 Diabetes (2020 Edition).

Variable	CKD without T2DM (*n* = 158)			CKD with T2DM (*n* = 214)		
	MetS− (*n* = 79)	MetS+ (*n* = 79)	*p*-Value	MetS− (*n* = 42)	MetS+ (*n* = 172)	*p*-Value
Age (years)	41.05 ± 14.86	45.58 ± 15.59	0.112	57.36 ± 8.36	53.56 ± 10.57	0.041
BMI (Kg/m^2^)	23.9 (21.6, 25.9)	27.3 (25.1, 29.8)	<0.001	22.85 (21.45, 25.63)	26.6 (24.46, 28.7)	<0.001
Waist circumference (cm)	81.33 ± 8.53	93.01 ± 9.33	<0.001	81 (75, 85.5)	90 (83, 95)	<0.001
Systolic blood pressure (mmHg)	124.8 ± 13.29	133.54 ± 14.29	<0.001	149.55 ± 24.67	152.99 ± 22.83	0.371
Diastolic blood pressure (mmHg)	74.82 ± 9.49	80.72 ± 11.09	0.001	84.74 ± 12.41	89.52 ± 12.64	0.041
FBG (mmol/L)	4.48 (4.17, 4.74)	4.56 (4.3, 5.45)	0.015	5.32 (4.46, 6.71)	5.61 (4.69, 6.89)	0.463
TC (mmol/L)	4.21 (3.49, 4.83)	4.19 (3.65, 5.18)	0.688	3.94 (3.31, 4.99)	4.44 (3.58, 5.61)	0.463
TG (mmol/L)	1.37 (1.07, 1.75)	2.41 (1.91, 3.22)	<0.001	1.17 (0.95, 1.32)	2.06 (1.55, 2.96)	<0.001
HDL-C (mmol/L)	1.16 (0.93, 1.41)	0.86 (0.76, 0.98)	<0.001	1.24 (1.12, 1.39)	0.88 (0.75, 1.02)	<0.001
LDL-C (mmol/L)	2.75 (2.15, 3.14)	2.55 (1.98, 3.22)	0.279	2.53 (1.91, 3.53)	2.62 (1.97, 3.48)	0.706
eGFR, mL/min per 1.73 m^2^	55.75 (35.84, 95.25)	43.59 (26.86, 61.16)	0.003	34.85 (18.33, 69.97)	34.96 (15.86, 64.71)	0.853
Hemoglobin (g/L)	127.15 ± 20.55	131.43 ± 22.45	0.213	115.36 ± 22.35	122.39 ± 25.146	0.099
Creatinine (mmol/L)	107.6 (80.9, 159.3)	135.8 (107.1, 192)	0.003	147.15 (81.83, 275.2)	159.55 (94.68, 285.93)	0.753
Uric acid (mmol/L)	404.28 ± 100.38	434.56 ± 113.14	0.077	381.28 ± 99.64	420.95 ± 113.17	0.038
Hypertension, *n* (%)	38 (48.1)	64 (81)	<0.001	33 (78.6)	162 (94.2)	0.004
Cardiovascular disease, *n* (%)	9 (11.4)	8 (10.1)	1	17 (40.5)	63 (36.6)	0.385
VFA (cm^2^)	79.54 ± 30.23	134.58 ± 41.27	<0.001	64 (43, 99)	119 (90, 148)	<0.001
SFA (cm^2^)	174 (130, 204)	235 (205, 276)	<0.001	141 (115, 184)	204 (168, 239)	<0.001
VAI	1.5 (1.04, 2.13)	3.48 (2.75, 4.64)	<0.001	0.99 (0.88, 1.39)	2.97 (1.98, 4.89)	<0.001
CVAI	69.26 ± 38.33	130.06 ± 38.94	<0.001	80.34 (50.96, 104.24)	120.36 (94.43, 143.42)	<0.001
LAP	24.65 (12.06, 33.75)	67.41 (47.32, 93.21)	<0.001	18.53 (10.71, 23.98)	46.38 (30.77, 81)	<0.001

BMI, body mass index; FBG, fasting blood glucose; TC, total cholesterol; TG, triglycerides; HDL-C, high-density lipoprotein cholesterol; LDL-C, low-density lipoprotein cholesterol; eGFR, estimated glomerular filtration rate; VFA, visceral fat area; SFA, subcutaneous fat area; VAI, visceral adiposity index; CVAI, Chinese visceral adiposity index; LAP, lipid accumulation product; MetS, metabolic syndrome; CKD, chronic kidney disease; T2DM, Type 2 diabetes mellitus; Data are mean (SD) or median (interquartile range), unless otherwise stated.

**Table 2:** Comparison of clinical characteristics of female patients according to the Chinese Guidelines for the Prevention and Treatment of Type 2 Diabetes (2020 Edition).

Variable	CKD without T2DM (*n* = 95)			CKD with T2DM (*n* = 70)		
	MetS− (*n* = 69)	MetS+ (*n* = 26)	*p*-Value	MetS− (*n* = 11)	MetS+ (*n* = 59)	*p*-Value
Age (years)	46.26 ± 11.89	49.35 ± 13.28	0.169	56.82 ± 10.69	58.86 ± 9.11	0.462
BMI (Kg/m^2^)	22.91 ± 3.78	26.16 ± 3.94	0.001	23.31 ± 2.65	26.06 ± 3.75	0.03
Waist circumference (cm)	76 (70, 81.5)	88.5 (80.75, 91.25)	<0.001	77.64 ± 6.31	87.19 ± 7.95	<0.001
Systolic blood pressure (mmHg)	119 (108, 135)	129 (118.5, 139)	0.048	134.55 ± 23.36	151.9 ± 24.07	0.026
Diastolic blood pressure (mmHg)	76 (68.5, 83.5)	78 (71.5, 88.25)	0.185	77.64 ± 12.53	85.63 ± 11.69	0.026
FBG (mmol/L)	4.43 (4.03, 4.78)	4.57 (4.13, 4.87)	0.339	5.35 (4.79, 7.03)	5.91 (4.94, 7.1)	0.508
TC (mmol/L)	4.85 (4.15, 5.59)	4.34 (3.44, 5.15)	0.021	4.2 (3.77, 4.83)	5.11 (4.07, 6.17)	0.11
TG (mmol/L)	1.53 (1.17, 2.17)	2.39 (1.81, 3.39)	<0.001	1.2 (1, 1.64)	2.43 (1.83, 3.72)	<0.001
HDL-C (mmol/L)	1.37 (1.17, 1.65)	0.91 (0.87, 1.09)	<0.001	1.26 (1.07, 1.54)	1.04 (0.85, 1.18)	0.006
LDL-C (mmol/L)	2.95 (2.48, 3.67)	2.69 (1.74, 3.45)	0.072	2.71 (2.14, 3.23)	3.02 (2.1, 3.96)	0.366
eGFR, mL/min per 1.73 m^2^	53.43 (31.44, 95.07)	33.76 (24.07, 51.46)	0.005	70.41 ± 32.05	59 ± 33.59	0.313
Hemoglobin (g/L)	112.58 ± 15.83	108.12 ± 16.47	0.229	117.09 ± 18.43	115.71 ± 20.86	0.838
Creatinine (mmol/L)	112.2 (68.5, 165.65)	159.75 (116.55, 215.63)	0.008	89.9 (59.1, 128.5)	103.5 (70.8, 156.3)	0.415
Uric acid (mmol/L)	349.19 ± 96.79	382.84 ± 92.22	0.129	348 (233.4, 385.6)	358.6 (287.7, 441.1)	0.255
Hypertension, *n* (%)	40 (58)	23 (88.5)	0.007	6 (54.5)	53 (89.8)	0.011
Cardiovascular disease, *n*(%)	5 (7.2)	3 (11.5)	0.679	1 (9.1)	18 (30.5)	0.267
VFA (cm^2^)	66.22 ± 27.63	92.19 ± 31.32	<0.001	51 (34, 82)	99 (86.5, 119)	<0.001
SFA (cm^2^)	149 (102.5, 199.5)	217.5 (162.75, 263.75)	<0.001	151.45 ± 56.73	204.26 ± 60.57	0.007
VAI	2.12 (1.36, 2.76)	4.18 (3.16, 6.36)	<0.001	1.69 (1.3, 2.35)	4.41 (2.69, 7.23)	<0.001
CVAI	68.55 ± 39.82	111.12 ± 34.13	<0.001	85.61 ± 24.98	125.94 ± 27.93	<0.001
LAP	28.88 (16.95, 43.88)	72.17 (46.96, 90.56)	<0.001	26.5 (13, 47.12)	72.9 (46.6, 103.04)	<0.001

BMI, body mass index; FBG, fasting blood glucose; TC, total cholesterol; TG, triglycerides; HDL-C, high-density lipoprotein cholesterol; LDL-C, low-density lipoprotein cholesterol; eGFR, estimated glomerular filtration rate; VFA, visceral fat area; SFA, subcutaneous fat area; VAI, visceral adiposity index; CVAI, Chinese visceral adiposity index; LAP, lipid accumulation product; MetS, metabolic syndrome; CKD, chronic kidney disease; T2DM, Type 2 diabetes mellitus; Data are mean (SD) or median (interquartile range), unless otherwise stated.

**Table 3:** Predictive value of seven obesity- and lipid-related indices in China (2020) criteria and multivariate logistic regression analysis.

Variable	Optimal Cut-Offs	Youden Index	Sensitivity (%)	Specificity (%)	OR (95% CI)	*p*-Value
**CKD with T2DM** **(Men)**						
BMI	23.6	0.4723	83.82	63.41	1.378 (1.187–1.600)	<0.001
WC	87	0.5048	61.3	89.2	1.160 (1.086–1.240)	<0.001
VFA (cm^2^)	101	0.5053	63.4	87.2	1.021 (1.010–1.032)	<0.001
SFA (cm^2^)	149	0.4539	83.85	61.54	1.019 (1.010–1.028)	<0.001
VAI	1.51	0.7802	86.84	91.18	40.585 (8.683–189.695)	<0.001
CVAI	111.21	0.5956	62.5	97.06	1.050 (1.030–1.071)	<0.001
LAP	31.44	0.7182	74.68	97.14	1.145 (1.083–1.209)	<0.001
**CKD with T2DM** **(Women)**						
BMI	22.7	0.3421	79.66	54.55	1.320 (1.029–1.694)	0.029
WC	81	0.6106	79.25	81.82	1.226 (1.070–1.405)	0.003
VFA (cm^2^)	88	0.6284	71.93	90.91	1.066 (1.025–1.109)	0.001
SFA (cm^2^)	159	0.4992	77.19	72.73	1.020 (1.004–1.035)	0.012
VAI	1.806	0.7084	98.11	72.73	5.076 (1.247–20.657)	0.023
CVAI	117.78	0.6415	64.15	100	1.069 (1.027–1.114)	0.001
LAP	56.76	0.6415	64.15	100	1.100 (1.030–1.175)	0.004
**CKD without T2DM** **(Men)**						
BMI	26.9	0.4684	56.96	89.87	1.441 (1.247–1.664)	<0.001
WC	89	0.5823	69.62	88.61	1.186 (1.114–1.262)	<0.001
VFA (cm^2^)	113	0.6329	72.15	91.14	1.051 (1.033–1.070)	<0.001
SFA (cm^2^)	208	0.5316	74.68	78.48	1.027 (1.017–1.037)	<0.001
VAI	2.35	0.6962	83.54	86.08	7.514 (3.757–15.027)	<0.001
CVAI	113.09	0.6329	73.42	89.87	1.055 (1.036–1.075)	<0.001
LAP	39.82	0.7089	86.08	84.81	1.137 (1.083–1.193)	<0.001
**CKD without T2DM** **(Women)**						
BMI	25.4	0.4365	65.38	78.26	1.305 (1.121–1.520)	0.001
WC	84	0.5474	69.23	85.51	1.127 (1.058–1.200)	<0.001
VFA (cm^2^)	71	0.3924	76.92	62.32	1.037 (1.015–1.060)	0.001
SFA (cm^2^)	201	0.4509	65.38	79.71	1.011 (1.005–1.018)	0.001
VAI	3.11	0.6844	84.62	83.82	3.008 (1.789–5.056)	<0.001
CVAI	75.708	0.4581	88.46	57.35	1.056 (1.029–1.084)	<0.001
LAP	34.92	0.6042	92.31	68.12	1.103 (1.054–1.154)	<0.001

Adjusted for age, systolic blood pressure, total cholesterol, LDL, eGFR; 95% CI,95% confidence interval; BMI, body mass index; WC, waist circumference; VFA, visceral fat area; SFA, subcutaneous fat area; VAI, visceral adiposity index; CVAI, Chinese visceral adiposity index; LAP, lipid accumulation product; CKD, chronic kidney disease; T2DM, Type 2 diabetes mellitus; OR, odds ratio.

**Table 4:** Area under the curve of seven obesity- and lipid-related indices under different metabolic syndrome criteria.

Variable	MetS-China (2020) Criterion		MetS-NCEP-ATPIII Criterion		MetS-IDF Criterion	
	AUC (95% CI)	*p*-Value	AUC (95% CI)	*p*-Value	AUC (95% CI)	*p*-Value
**CKD with T2DM** **(Men)**						
BMI	0.782 (0.740–0.876)	<0.001	0.686 (0.597–0.776)	<0.001	0.897 (0.855–0.94)	<0.001
WC	0.808 (0.740–0.876)	<0.001	0.694 (0.610–0.778)	<0.001	0.998 (0.995–1)	<0.001
VFA (cm^2^)	0.785 (0.706–0.864)	<0.001	0.707 (0.622–0.792)	<0.001	0.915 (0.874–0.956)	<0.001
SFA (cm^2^)	0.750 (0.652–0.847)	<0.001	0.663 (0.569–0.757)	0.001	0.928 (0.893–0.963)	<0.001
VAI	0.920 (0.864–0.976)	<0.001	0.957 (0.919–0.995)	<0.001	0.695 (0.619–0.771)	<0.001
CVAI	0.847 (0.785–0.908)	<0.001	0.765 (0.691–0.838)	<0.001	0.973 (0.953–0.992)	<0.001
LAP	0.902 (0.857–0.946)	<0.001	0.858 (0.806–0.911)	<0.001	0.860 (0.808–0.912)	<0.001
**CKD with T2DM** **(Women)**						
BMI	0.715 (0.555–0.876)	0.026	0.744 (0.610–0.879)	0.036	0.906 (0.820–0.993)	<0.001
WC	0.839 (0.708–0.970)	<0.001	0.807 (0.676–0.938)	0.008	1(1–1)	<0.001
VFA (cm^2^)	0.860 (0.734–0.987)	<0.001	0.882 (0.784–0.981)	0.001	0.971 (0.936–1)	<0.001
SFA (cm^2^)	0.754 (0.586–0.921)	0.008	0.773 (0.639–0.907)	0.019	0.971 (0.928–1)	<0.001
VAI	0.902 (0.8–1)	<0.001	0.965 (0.912–1)	<0.001	0.760 (0.604–0.916)	0.003
CVAI	0.878 (0.782–0.975)	<0.001	0.862 (0.752–0.972)	0.002	0.913 (0.832–0.993)	<0.001
LAP	0.885 (0.794–0.976)	<0.001	0.865 (0.768–0.962)	0.002	0.890 (0.776–1)	<0.001
**CKD without T2DM** **(Men)**						
BMI	0.798 (0.729–0.867)	<0.001	0.704 (0.621–0.786)	<0.001	0.917 (0.875–0.959)	<0.001
WC	0.830 (0.765–0.894)	<0.001	0.717 (0.635–0.789)	<0.001	0.964 (0.937–0.991)	<0.001
VFA (cm^2^)	0.865 (0.807–0.922)	<0.001	0.741 (0.662–0.820)	<0.001	0.947 (0.915–0.979)	<0.001
SFA (cm^2^)	0.820 (0.756–0.885)	<0.001	0.727 (0.648–0.806)	<0.001	0.903 (0.857–0.949)	<0.001
VAI	0.910 (0.866–0.955)	<0.001	0.895 (0.844–0.945)	<0.001	0.776 (0.703–0.849)	<0.001
CVAI	0.879 (0.826–0.931)	<0.001	0.773 (0.7–0.847)	<0.001	0.980 (0.963–0.998)	<0.001
LAP	0.921 (0.881–0.961)	<0.001	0.848 (0.789–0.907)	<0.001	0.909 (0.863–0.954)	<0.001
**CKD without T2DM** **(Women)**						
BMI	0.731 (0.618–0.844)	0.001	0.751 (0.648–0.855)	<0.001	0.867 (0.794–0.940)	<0.001
WC	0.793 (0.689–0.897)	<0.001	0.778 (0.678–0.877)	<0.001	0.923 (0.868–0.978)	<0.001
VFA (cm^2^)	0.734 (0.617–0.852)	<0.001	0.679 (0.562–0.796)	0.005	0.851 (0.773–0.930)	<0.001
SFA (cm^2^)	0.741 (0.633–0.850)	<0.001	0.734 (0.630–0.839)	<0.001	0.890 (0.826–0.955)	<0.001
VAI	0.881 (0.812–0.949)	<0.001	0.921 (0.869–0.973)	<0.001	0.824 (0.741–0.908)	<0.001
CVAI	0.782 (0.684–0.881)	<0.001	0.835 (0.753–0.918)	<0.001	0.896 (0.833–0.960)	<0.001
LAP	0.854 (0.769–0.939)	<0.001	0.891 (0.821–0.960)	<0.001	0.905 (0.845–0.965)	<0.001

BMI, body mass index; WC, waist circumference; VFA, visceral fat area; SFA, subcutaneous fat area; VAI, visceral adiposity index; CVAI, Chinese visceral adiposity index; LAP, lipid accumulation product; OR, odds ratio; CKD, chronic kidney disease; T2DM, Type 2 diabetes mellitus; MetS-NCEP-ATP III, Metabolic syndrome diagnosis according to the revised National Cholesterol Education Program Adult Treatment Group; MetS-IDF, Metabolic syndrome diagnosis according to the International Diabetes Federation.

## Data Availability

The data presented in this study are available upon request from the corresponding author.

## Supplementary Materials

The following supporting information can be downloaded at: https://www.mdpi.com/article/10.3390/nu14071334/s1. Table S1. Predictive value and multivariate logistic regression analysis of seven obesity- and lipid-related indices in the NCEP-ATP III criteria; Table S2. Predictive value of seven obesity- and lipid-related indices in IDF criteria and multivariate logistic regression analysis; Figure S1. Flowchart of selection process for the data included in this analysis. WC, waist circumferences; VFA, visceral fat area; Figure S2. Comparison of diagnostic values of BMI, WC, VFA, SFA, VAI, CVAI and LAP in predicting metabolic syndrome using NECP-ATPIII criteria in CKD men and women with T2DM and without T2DM; Figure S3. Comparison of diagnostic values of BMI, WC, VFA, SFA, VAI, CVAI and LAP in predicting metabolic syndrome using IDF criteria in CKD men and women with and without T2DM.
