# Supplementary material for: Optimal Obesity- and Lipid-Related Indices for Predicting Metabolic Syndrome in Chronic Kidney Disease Patients with and without Type 2 Diabetes Mellitus in China
Source: Nutrients. 2022 Mar 23;14(7):1334. doi: 10.3390/nu14071334 (PMC9002364; doi:10.3390/nu14071334)
Supplement: Supplementary file 1 [file nutrients-14-01334-s001.zip › nutrients-1596639-supplementary.pdf]

**Supplementary Table S1**. Predictive value and multivariate logistic regression analysis of seven obesity and lipid-related indices in NCEP-ATPⅢ criteria

| Variable | Optimal cut-offs | Youden Index | Sensitivity（%） | Specificity（%） | OR（95% CI） | P-value |
|---|---|---|---|---|---|---|
| **CKD with T2DM (Men)** | | | | | | |
| BMI | 24.4 | 0.3194 | 75.8 | 56.14 | 1.196（1.075-1.332） | 0.001 |
| WC | 87 | 0.3115 | 60 | 71.15 | 1.089（1.039-1.141） | < 0.001 |
| VFA ($cm^2$) | 101 | 0.3524 | 63.01 | 72.22 | 1.014（1.006-1.023） | 0.001 |
| SFA ($cm^2$) | 149 | 0.3171 | 83.5 | 48.15 | 1.011（1.005-1.018） | 0.001 |
| VAI | 1.58 | 0.8439 | 90.51 | 93.88 | 71.795（14.460-356.467） | < 0.001 |
| CVAI | 111.21 | 0.4237 | 62.77 | 79.59 | 1.033（1.019-1.046） | < 0.001 |
| LAP | 34.84 | 0.5722 | 71.22 | 86 | 1.105（1.064-1.146） | < 0.001 |
| **CKD with T2DM (Women)** | | | | | | |
| BMI | 25 | 0.5556 | 55.56 | 100 | 1.478（1.013-2.156） | 0.043 |
| WC | 77 | 0.5739 | 85.96 | 71.43 | 1.307（1.046-1.632） | 0.018 |
| VFA ($cm^2$) | 86 | 0.7377 | 73.77 | 100 | 1.154（1.012-1.317） | 0.033 |
| SFA ($cm^2$) | 200 | 0.5410 | 54.1 | 100 | 1.023（1.003-1.044） | 0.026 |
| VAI | 1.69 | 0.8396 | 98.25 | 85.71 | 1.749（1.083-2.824） | 0.0223 |
| CVAI | 97.801 | 0.6992 | 84.21 | 85.71 | 1.169（1.026-1.333） | 0.019 |
| LAP | 47.12 | 0.7193 | 71.93 | 100 | 1.033（1.005-1.061） | 0.0208 |
| **CKD without T2DM (Men)** | | | | | | |
| BMI | 26.9 | 0.3081 | 52.46 | 78.35 | 1.239（1.101-1.393） | < 0.001 |
| WC | 87 | 0.3502 | 72.13 | 62.89 | 1.101（1.049-1.155） | < 0.001 |
| VFA ($cm^2$) | 119 | 0.4210 | 60.66 | 81.44 | 1.026（1.014-1.038） | < 0.001 |
| SFA ($cm^2$) | 208 | 0.3914 | 72.13 | 67.01 | 1.017（1.009-1.025） | < 0.001 |
| VAI | 2.702 | 0.6978 | 85.25 | 84.54 | 4.055（2.392-6.874） | < 0.001 |

| | | | | | | |
|---|---|---|---|---|---|---|
| CVAI | 112.25 | 0.4205 | 68.85 | 73.2 | 1.031 (1.017-1.044) | < 0.001 |
| LAP | 39.82 | 0.5638 | 85.25 | 71.13 | 1.058 (1.035-1.082) | < 0.001 |
| **CKD without T2DM (Women)** | | | | | | |
| BMI | 23 | 0.4157 | 81.25 | 60.32 | 1.378 (1.169-1.624) | < 0.001 |
| WC | 86 | 0.4673 | 56.25 | 90.48 | 1.123 (1.055-1.195) | < 0.001 |
| VFA ($cm^2$) | 74 | 0.3070 | 65.62 | 65.08 | 1.023 (1.005-1.042) | 0.014 |
| SFA ($cm^2$) | 210 | 0.3884 | 53.13 | 85.71 | 1.012 (1.005-1.019) | 0.001 |
| VAI | 3.11 | 0.6996 | 81.25 | 88.71 | 6.322 (2.665-14.996) | < 0.001 |
| CVAI | 90.71 | 0.5393 | 78.12 | 75.81 | 1.076 (1.041-1.112) | < 0.001 |
| LAP | 44.24 | 0.6850 | 84.37 | 84.13 | 1.160 (1.083-1.243) | < 0.001 |

Adjusted for age, systolic blood pressure, total cholesterol, LDL, eGFR；95% CI，95% confidence interval；BMI, body mass index；WC, waist circumference；VFA, visceral fat area；SFA, subcutaneous fat area；VAI, visceral adiposity index；CVAI，Chinese visceral adiposity index；LAP，Lipid accumulation product；OR, odds ratio;

**Supplementary Table S2.** Predictive value of seven obesity and lipid-related indices in IDF criteria and multivariate logistic regression analysis

| Variable | Optimal cut-offs | Youden Index | Sensitivity (%) | Specificity (%) | OR (95% CI) | P-value |
|---|---|---|---|---|---|---|
| **CKD with T2DM (Men)** | | | | | | |
| BMI | 25.9 | 0.6216 | 87.36 | 74.80 | 2.031 (1.646-2.507) | < 0.001 |
| WC | 89 | 0.991 | 100 | 99.1 | 2.733 (1.797-4.156) | < 0.001 |
| VFA ($cm^2$) | 123 | 0.6788 | 78.05 | 89.93 | 1.047 (1.032-1.062) | < 0.001 |
| SFA ($cm^2$) | 196 | 0.7234 | 92.68 | 79.66 | 1.064 (1.043-1.085) | < 0.001 |
| VAI | 2.62 | 0.34 | 65.43 | 68.57 | 1.160 (0.977-1.377) | 0.091 |
| CVAI | 114.94 | 0.8801 | 97.53 | 90.48 | 1.197 (1.119-1.280) | < 0.001 |
| LAP | 39.79 | 0.5710 | 83.95 | 73.15 | 1.055 (1.036-1.075) | < 0.001 |
| **CKD with T2DM (Women)** | | | | | | |
| BMI | 22.7 | 0.7481 | 92.45 | 82.35 | 2.999 (1.653-5.441) | < 0.001 |

| | | | | | | |
|---|---|---|---|---|---|---|
| WC | 79 | 1 | 100 | 100 | 1.206（1.085-1.340） | < 0.001 |
| VFA (cm$^2$) | 81 | 0.8579 | 92.45 | 93.33 | 1.104（1.048-1.164） | < 0.001 |
| SFA (cm$^2$) | 143 | 0.8956 | 96.23 | 93.33 | 1.109（1.034-1.189） | 0.004 |
| VAI | 3.25 | 0.5171 | 66 | 85.71 | 1.484（1.067-2.064） | 0.019 |
| CVAI | 97.801 | 0.7057 | 92 | 78.57 | 1.168（1.059-1.288） | 0.002 |
| LAP | 47.12 | 0.7286 | 80 | 92.86 | 1.107（1.042-1.177） | 0.001 |
| **CKD without T2DM (Men)** | | | | | | |
| BMI | 26 | 0.7062 | 88.89 | 81.73 | 2.146（1.672-2.756） | < 0.001 |
| WC | 89 | 0.9038 | 100 | 90.38 | 1.673（1.384-2.023） | < 0.001 |
| VFA (cm$^2$) | 114 | 0.7728 | 90.74 | 86.54 | 1.088（1.057-1.119） | < 0.001 |
| SFA (cm$^2$) | 225 | 0.6884 | 85.19 | 83.65 | 1.048（1.031-1.066） | < 0.001 |
| VAI | 2.74 | 0.5004 | 74.07 | 75.96 | 1.472（1.199-1.807） | < 0.001 |
| CVAI | 113.98 | 0.8853 | 98.15 | 90.38 | 1.211（1.111-1.320） | < 0.001 |
| LAP | 45.6 | 0.7062 | 88.89 | 81.73 | 1.070（1.046-1.095） | < 0.001 |
| **CKD without T2DM (Women)** | | | | | | |
| BMI | 25.4 | 0.6282 | 76.67 | 86.15 | 1.619（1.317-1.990） | < 0.001 |
| WC | 79 | 0.8462 | 100 | 84.62 | 1.239（1.123-1.367） | < 0.001 |
| VFA (cm$^2$) | 71 | 0.6077 | 90 | 70.77 | 1.057（1.030-1.085） | < 0.001 |
| SFA (cm$^2$) | 175 | 0.6538 | 90 | 75.38 | 1.022（1.012-1.032） | < 0.001 |
| VAI | 2.64 | 0.6188 | 90 | 71.87 | 1.758（1.279-2.415） | < 0.001 |
| CVAI | 90.71 | 0.6969 | 90 | 79.69 | 1.108（1.058-1.160） | < 0.001 |
| LAP | 43.52 | 0.7795 | 93.33 | 84.62 | 1.147（1.081-1.217） | < 0.001 |

Adjusted for age, systolic blood pressure, total cholesterol, LDL, eGFR；95% CI，95% confidence interval；BMI, body mass index；WC, waist circumference；VFA, visceral fat area；SFA, subcutaneous fat area；VAI, visceral adiposity index；CVAI，Chinese visceral adiposity index；LAP，Lipid accumulation product；OR, odds ratio;

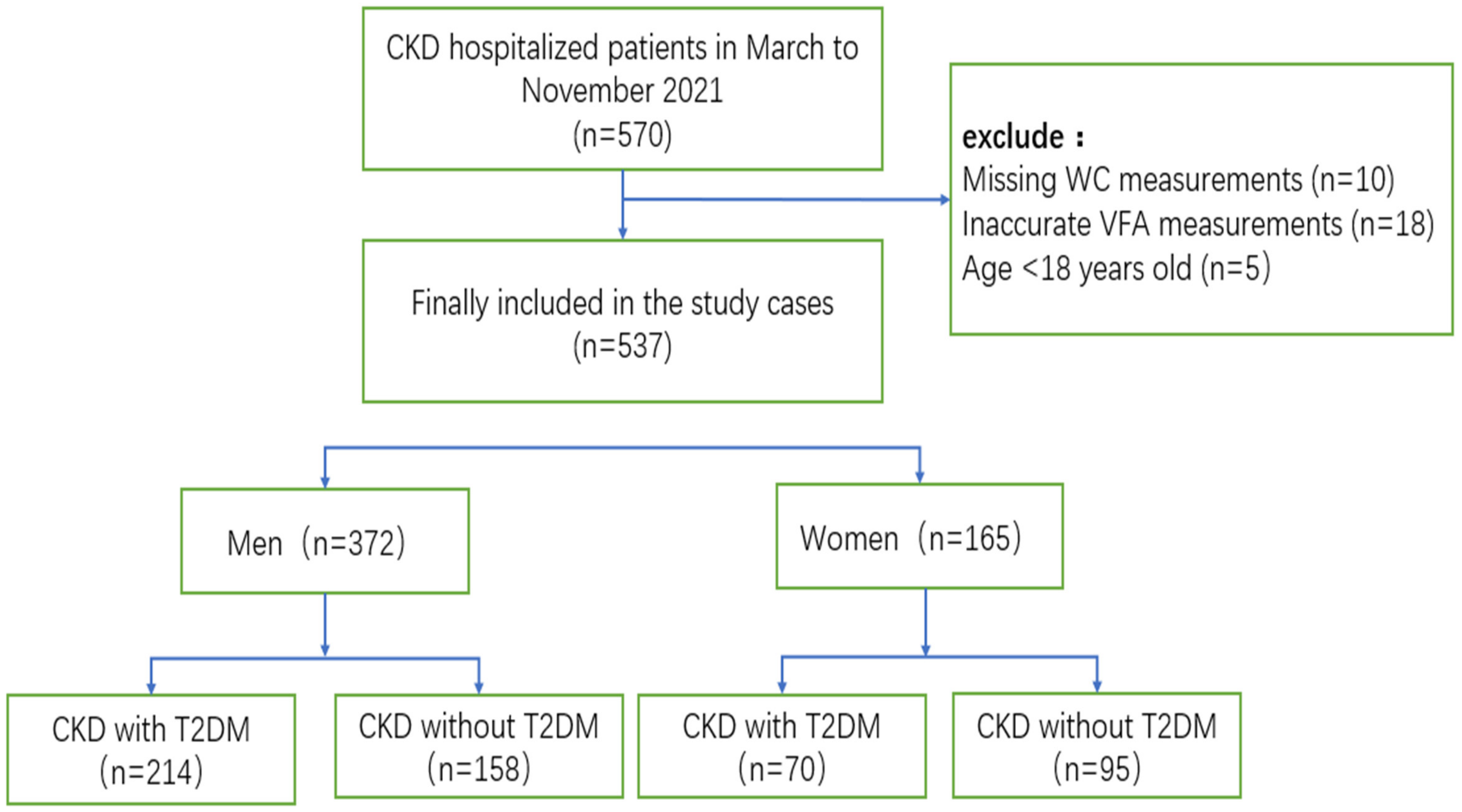


**Supplementary Figure S1**. Flowchart of selection process for the data included in this analysis. WC, waist circumferences; VFA, visceral fat area

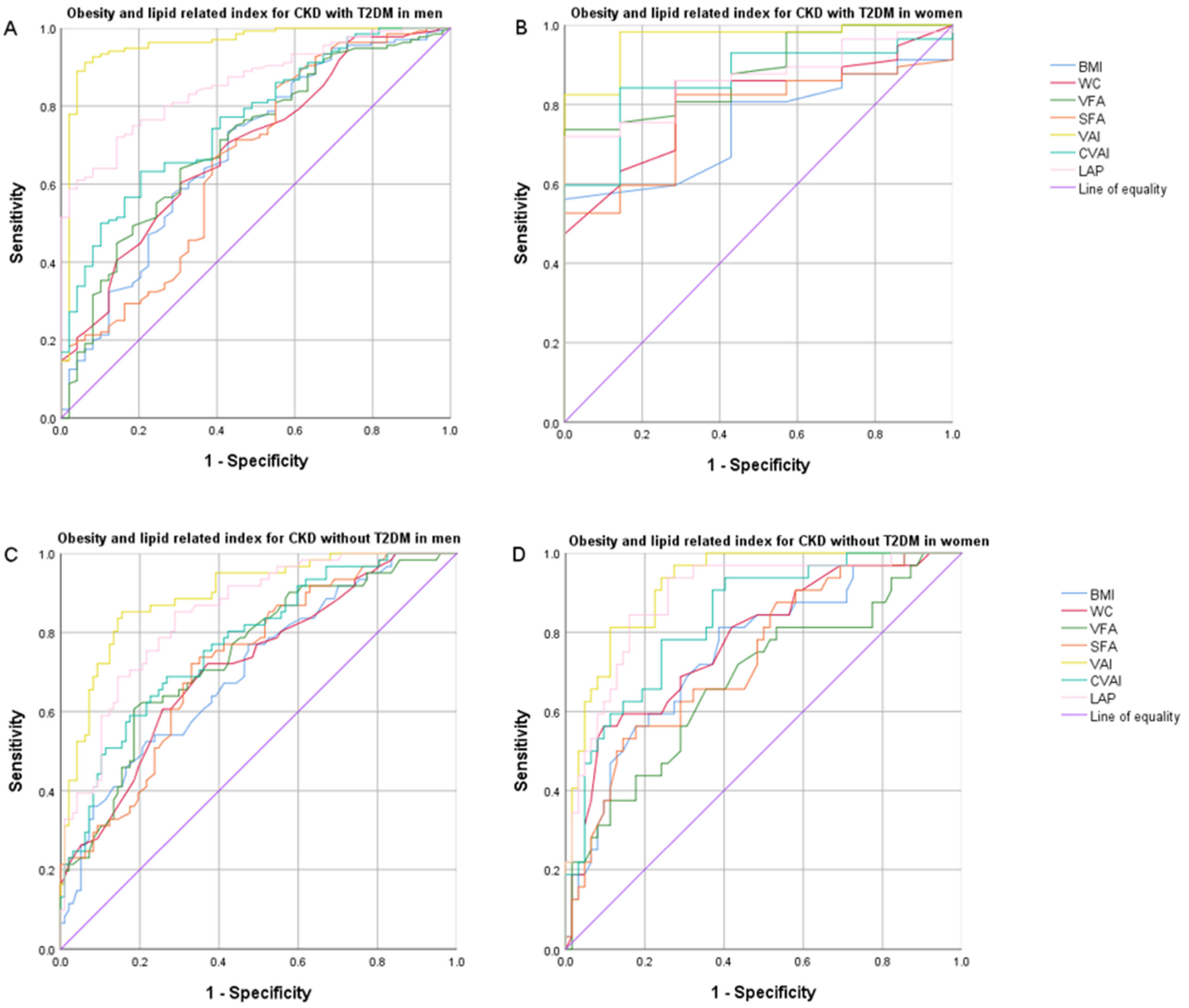


**Supplementary Figure S2.** Comparison of diagnostic value of BMI, WC, VFA, SFA, VAI, CVAI and LAP in predicting metabolic syndrome using NECP-ATP III criteria in CKD males and females with T2DM and without T2DM.

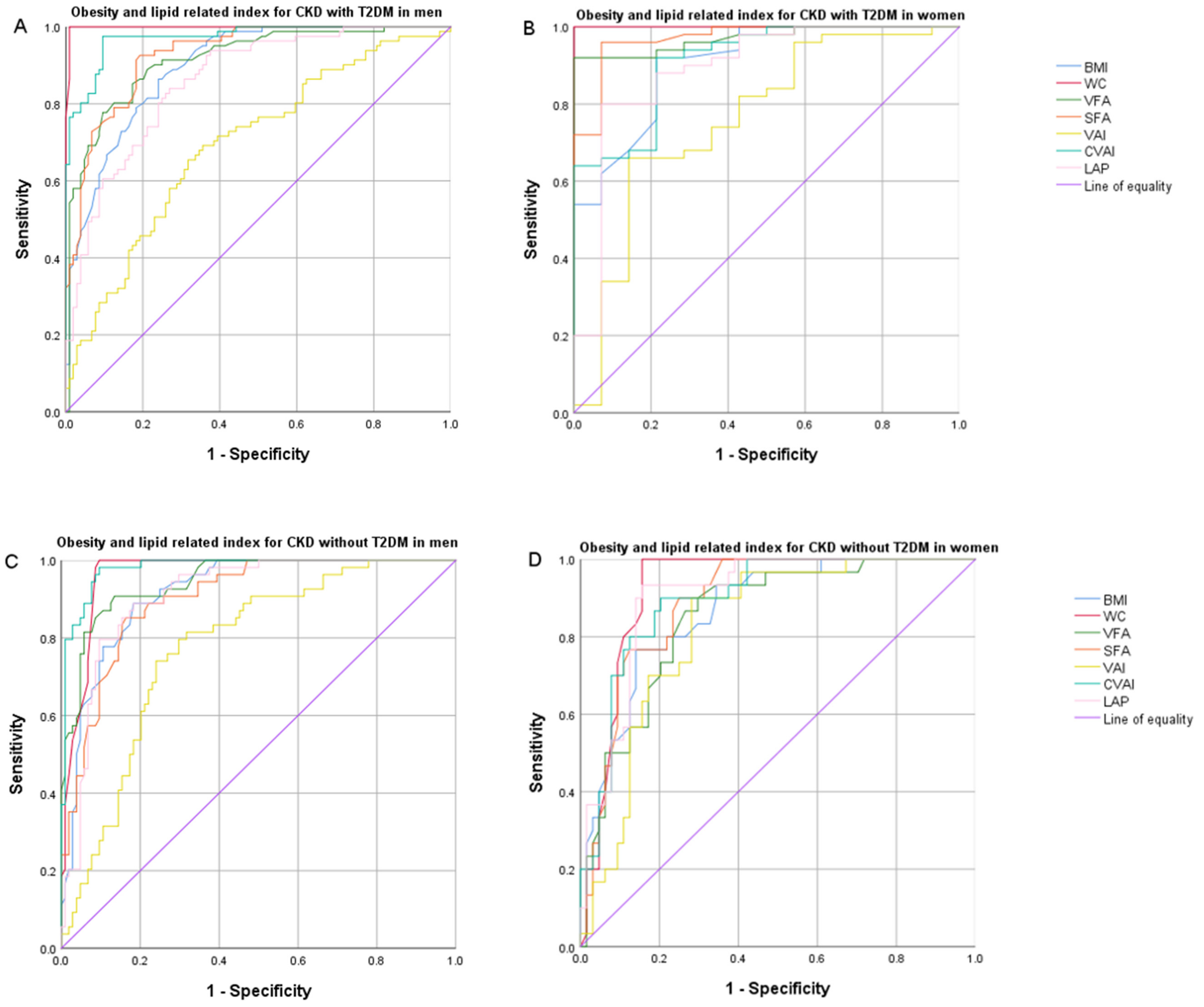


**Supplementary Figure S3.** Comparison of diagnostic value of BMI, WC, VFA, SFA, VAI, CVAI, and LAP in predicting metabolic syndrome using IDF criteria in CKD men and women with T2DM and without T2DM.
